# Smoking Functions as a Negative Regulator of IGF1 and Impairs Adipokine Network in Patients with Rheumatoid Arthritis

**DOI:** 10.1155/2016/3082820

**Published:** 2016-03-03

**Authors:** Malin C. Erlandsson, Roberto Doria Medina, Sofia Töyrä Silfverswärd, Maria I. Bokarewa

**Affiliations:** Department of Rheumatology and Inflammation Research, Sahlgrenska Medical Academy, Gothenburg University, 40583 Gothenburg, Sweden

## Abstract

*Objectives.* Smoking is pathogenic for rheumatoid arthritis (RA) being tightly connected to the genetic and serological risk factors for this disease. This study aims to understand connections between cigarette smoking and serum levels of IGF1 and adipokines in RA.* Methods.* Serum levels of IGF1 and adipokines leptin, adiponectin, resistin, and visfatin were measured in two independent cohorts of RA patients from Gothenburg (*n* = 350) and Leiden (*n* = 193). An association of these parameters with smoking was tested in a direct comparison and proved by bivariate correlation analysis. The obtained associations were further tested in multivariate regression models where the confounders (age, gender, disease duration, and BMI) were controlled.* Results.* The smokers had significantly lower serum levels of IGF1, adiponectin, and leptin compared to never smokers. In regression analysis, smoking and low leptin, but not adiponectin, were associated and predicted low IGF1. Additionally, high disease activity and high BMI increased the probability of low leptin.* Conclusions.* The study indicates cigarette smoking as an important cause of a relative IGF1 and leptin deficiency in RA patients. This novel association between smoking and hypoleptinemia may be of importance for long-term prognosis of RA and for prediction of comorbidities.

## 1. Introduction

Smoking is known for its negative impact on health being tightly connected to early ageing, carcinogenicity, high risk of lung and cardiovascular diseases, and early mortality [1]. Extensive research in recent years indicates a tight connection between cigarette smoking and rheumatoid arthritis (RA). The prevalence of cigarette smoking appears to be higher in patients with RA [2]. At the preclinical and early stages of the disease smoking has been suggested to trigger the immune reactions controlled by HLA-DRB1 genes [3], which initiates joint inflammation and production of rheumatoid factor (RF) and antibodies to citrullinated peptides, classical serologic biomarkers of RA [4, 5]. In the established RA, cigarette smoking is related to the progressive joint damage, persistent disease activity, and development of rheumatic noduli [6, 7]. These reports are met by controversy from other studies, which reported a lower radiographic disease progression and no significant effects on disease activity in the smoking patients with RA [8–10]. In addition, nicotine exposure through smokeless tobacco was not associated with risk of RA [11]. Thus, the molecular events connecting cigarette smoking to severe joint damage and low efficacy of antirheumatic drugs indicates the role of combined biological mechanisms triggered by smoking during arthritis.

Smoking is inversely associated with circulating levels of IGF1 indicating a direct inhibitory effect [12, 13]. IGF1 is an important mediator in developmental processes including proliferation, growth, differentiation, and survival [14, 15]. Systemic and paracrine IGF1 deficiency is recognized by enhanced skeletal metabolism and progressive osteoporosis [16], development of glucose intolerance [17], premature atherosclerosis, and cardiovascular mortality [18]. Modern understanding of molecular mechanisms of IGF1 suppression by nicotine is connected with dysfunction of the hypothalamus-pituitary axis, where growth hormone, neuropeptide Y, and adipokine leptin have central role.

In human and in experimental RA, the alterations of the IGF1 system encompass the reduction of IGF1 levels [19, 20] and high expression of IGF1 binding proteins, which reduces bioavailability of IGF1 [20–22] despite the increased density of IGF1 receptors. The impairment of IGF1 system is viewed as a result of cytokine-driven chronic inflammation [23]. Clinically, RA patients with low levels of IGF1 have higher disease activity and are prone to cachexia [21]. However, the alleviation of inflammation with the antirheumatic treatment including corticosteroids and TNF inhibitors seldom restores IGF1 system in RA patients [22]. We hypothesise that the competitive binding and activation of the insulin/IGF1 receptor (IGF1R) by several members of the adipokine family including leptin, resistin, and visfatin [20, 24, 25] disturb normal function of IGF1 system in RA preventing positive feedback.

In RA, adipokines have been connected to the progressive joint destruction. Adiponectin and resistin levels were predictive of radiographic damage [26–28]. The levels of leptin showed a negative [29] and positive association with the radiographic damage [30]. Additionally, the baseline levels of adipokines may predict the effect of antirheumatic treatment [31, 32].

In the present study we asked if smoking was associated with the changes in IGF1 and could mediate the adipokine dependent mechanisms of inflammation in RA. The evaluation of two independent RA cohorts convincingly demonstrates that smoking is an essential factor contributing to low levels of IGF1 and leptin. This novel association between smoking, IGF1 and hypoleptinemia in RA may be of importance for long-term prognosis of the disease and for prediction of comorbidities.

## 2. Patients and Methods

### 2.1. Patient Material

570 patients from 2 independent RA cohorts (Gothenburg, *n* = 367, and Leiden, *n* = 203) were included in this cross-sectional observational study. Twenty-seven patients (4.7%, 17 patients of Gothenburg and 10 patients of the Leiden cohort) were excluded because of unknown smoking status or use of snuff resulting in 543 patients in total. Written informed consent was obtained from the participants.

The Göteborg cohort comprised the patients with rheumatoid arthritis who attended the Rheumatology Clinics at the Sahlgrenska University Hospital, Gothenburg, and the Rheumatology Unit of the Uddevalla Hospital and were current users of methotrexate. BMI, DAS28, and VAS-pain were recorded. The patients of Leiden cohort were randomly selected from the Leiden Early Arthritis Clinic cohort [33]. BMI, VAS-pain, and DAS44 were recorded. DAS44 was converted to DAS28 with a standardised formula. The study was approved by the Ethics Committee of Sahlgrenska University Hospital and the Leiden University Hospital.

The clinical characteristics of RA patients at enrolment are shown in Table 1. Patients were asked to report their smoking habits through a structured telephone interview or by filling in a questionnaire. Smoking was considered if the patients smoked or had smoked for the past year, based on self-report. Patients were divided by their smoking habits defined as current smokers, former smokers, or never smokers. Subjects who smoked previously and stopped smoking longer than 1 year before the study were considered former smokers.

### 2.2. Sampling and Storage

Blood samples were obtained from the cubital vein and centrifuged at 800 ×g for 15 min, aliquoted, and stored frozen at −70°C until use.

### 2.3. Laboratory Analyses

Biological markers were analysed by sandwich enzyme-linked immunosorbent assays (ELISAs) using matched pairs of specific antibodies and recombinant standards. Assays for human adiponectin (DY1065, 62 pg/mL), human leptin (DY398, 31 pg/mL), human resistin (DY1359, 10 pg/mL), and human free bioactive IGF1 (DY291, 4 pg/mL) were all purchased from RnD Systems (Minneapolis, MN, USA). Assays specific for human visfatin (AG-45A-0006TP-KI01; 125 pg/mL) were purchased from Adipogen, Inc. (Incheon, South Korea). All assays were performed according to the instructions of the manufacturers. ELISAs were read with a Spectramax 340 from Molecular Devices (Sunnyvale, CA, USA). The levels of rheumatoid factor (RF) antibodies were measured at the Clinical Immunology Laboratory of the Sahlgrenska University Hospital or Leiden Clinical Immunology Laboratory.

### 2.4. Statistics

Descriptive data are presented as the median, the interquartile range, the number, and the percentage. The material was stratified by smoking history into the patients who never smoked (NSm, *n* = 240), current smokers (Sm, *n* = 126), and former smokers (FSm, *n* = 177). Bivariate correlation between variables was examined by Spearman's correlation test. Differences between correlations were studied with Fisher's *r*-to-*z* test. The differences between groups were assessed by the Mann-Whitney *U* test or Kruskal Wallis test followed by Dunn's multiple comparisons test. The sensitivity and specificity of calculations were performed using 2 × 2 table analysis and Chi-square tests. To compensate for the absence of established cut-off points and to retain adequate statistic power, the material was categorized by tertiles within each cohort. For the binary logistic regression, the parameters were dichotomized by low compared to medium and high tertiles. Predictive value of smoking and adipokines for low levels of IGF1 and leptin was calculated by composing binary logistic regression models where low IGF1 or low leptin were chosen as the dependent variable and adipokines in tertiles, smoking habits, gender, and age as independent variables (*n* = 516). A second binary logistic regression set was performed where the clinical parameters disease duration, RF positivity, DAS28, VAS-pain, and BMI together with gender, age, and smoking habits, were added as independent variables (*n* = 246). All tests were two-tailed and conducted with 95% confidence. Statistical analyses were performed using Graphpad Prism v.6 and SPSS v.22 software.

## 3. Results

Clinical characteristics of the studied cohorts of RA patients are shown in Table 1. The Leiden cohort comprised patients with early RA and had lower disease duration (*p* < 0.001), higher disease activity (*p* < 0.001), higher VAS-pain (*p* < 0.001), and lower prevalence of RF-positive patients compared to the Gothenburg cohort of patients with established RA. The two cohorts were similar in gender composition and in BMI. Within the smoking groups, the Leiden cohort was sharply stratified into current smokers (Sm) and never smokers (NSm) (56%), while the Gothenburg cohort had a substantial proportion of former smokers (FSm) (43%). In both cohorts, Sm were more often men (Leiden, 46% men versus 22% women; Gothenburg, 23% men and 18% women), while FSm were older (*p* = 0.006), had longer disease duration (*p* < 0.001), and lower DAS28 (*p* = 0.05), and were more often RF-positive (*p* = 0.04) than NSm.

### 3.1. Effect of Smoking on the Serum Levels of IGF1 and Adipokines

**Figure 1:**
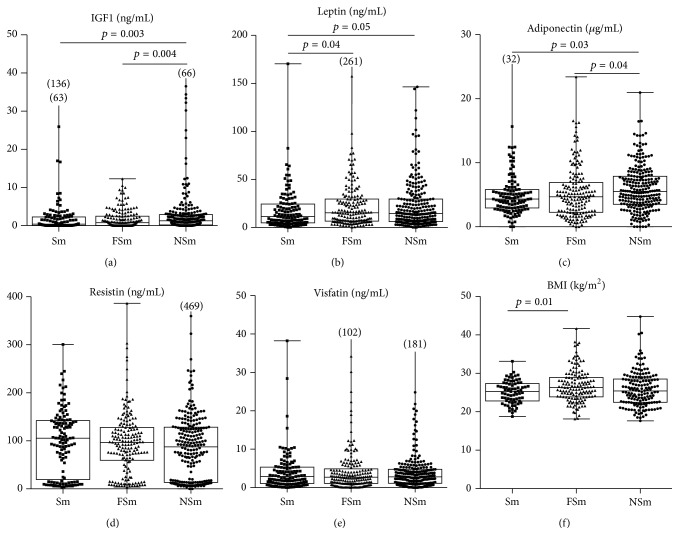
Serum levels of IGF1 and adipokines in RA patients grouped by smoking habits. Serum levels of IGF1 (a), leptin (b), resistin (d), adiponectin (c), visfatin (e), and BMI (f) in RA patients grouped by smoking habits in current smokers (Sm, *n* = 126), former smokers (FSm, *n* = 177), or never smokers (NSm, *n* = 240). Boxes represent median and interquartile range. The comparison was performed by the Kruskal Wallis nonparametric ANOVA with Dunn's multiple comparisons test.

**Figure 2:**
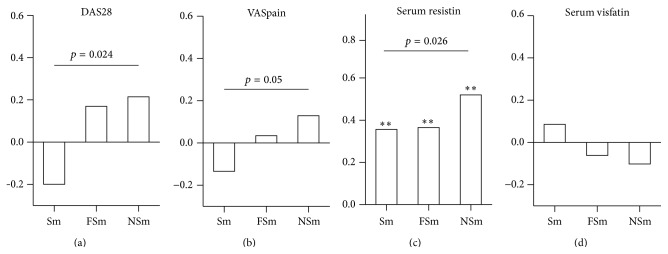
The correlations between serum levels of leptin and clinical parameters of RA in the smoking groups. The bars represent the correlation coefficients for the disease activity score (DAS28) (a), VAS-pain (b), and adipokine levels in serum (c, d) in RA patients grouped by smoking habits into smokers (Sm), former smokers (FSm), and never smokers (NSm). The correlations were analysed by the Spearman statistics. The Fisher's *r*-to-*z* test was used to compare the magnitude of the correlation coefficients between Sm and NSm. The *p* values below 0.01 are indicated ( ^*∗∗*^).

The highest serum levels of IGF1 were measured in NSm and they gradually declined with smoking. It was lowest in Sm, followed by FSm (Figure 1(a)). Additionally, Sm and FSm had higher probability to have IGF1 levels within lowest tertile compared to NSm (Sm: OR 2.24 [1.38–3.65], *p* = 0.0012; FSm: OR 1.85 [1.18–2.89], *p* = 0.0071). IGF1 is known to regulate the development and metabolism of adipose tissue. BMI, a surrogate measure of adipose tissue volume, showed no correlation with the IGF1 levels (rho = −0.053) and was similar between the smoking groups (Figure 1(f)). Correlations between BMI and adipokine levels are shown in Supplementary Table S1 (see Supplementary Material available online at http://dx.doi.org/10.1155/2016/3082820). Adipokines reflecting the metabolic activity of the fat tissue were affected by smoking. The levels of leptin and adiponectin were lower in Sm compared to NSm (Figures 1(b) and 1(c)), while the serum levels of resistin and visfatin were similar between the smoking groups. Bivariate correlation analyses revealed that NSm presented no or only a weak association between IGF1 and adipokine levels. In Sm, IGF1 was associated with leptin (rho = 0.233, *p* = 0.009) and resistin (rho = 0.210, *p* = 0.018). It was noted that smoking affected associations of leptin with clinical signs of RA. NSm had direct association between serum leptin and DAS28 (rho = 0.216), while in Sm this association became inverse (rho = −0.198). The difference in associations of leptin and DAS between Sm and NSm was significant (*p* = 0.024) (Figure 2(a)). Similar smoking dependent difference was seen in associations between leptin and VAS-pain (*p* = 0.056) (Figure 2(b)). The positive association between leptin and resistin was present in all smoking groups suggesting concordant effect of smoking on these parameters (Figure 2(c)).

### 3.2. Predictive Value of Smoking and Leptin for Low Levels of IGF1

To identify independent variables predicting low serum levels of IGF1, a binary logistic regression with backwards elimination was performed. In the first step of the model, the adipokine levels in tertiles, gender, age, and smoking groups were introduced. The importance of each variable was verified using Wald statistics and the variables of no importance (*p* > 0.1) were eliminated. The last step of the elimination analysis indicated that current smoking (OR 0.54 [95% CI 0.34–0.86], *p* = 0.009) and low levels of leptin (OR 0.73 [95% CI 0.57–0.93], *p* = 0.010) were predictive of low levels of IGF1. Adiponectin was eliminated from the regression model and showed no association with low IGF1 levels. The complete table of elimination steps is available in Supplementary Table S2.

In the second model, the clinical variables (DD, RF-positivity, DAS28, VAS-pain, and BMI) and the smoking groups were added to the analysis. The last step of the elimination demonstrated that present smoking (OR 0.39 [95% CI 0.18–0.84], *p* = 0.016) and high VAS-pain (OR 0.986 [95% CI 0.973–1.000], *p* = 0.044) were the independent variables to predict low IGF1 in RA patients (Supplementary Table S3).

Since low serum levels of leptin appeared to be an important predictor of low IGF1, a different binary logistic regression model was constructed to understand which clinical parameters were associated with the low levels of leptin. The disease duration, DAS28, age, smoking groups, RF-positivity, BMI, VAS-pain, and gender were included in the regression model. The final step of the backward elimination showed that high DAS28 (OR 0.82 [95% CI 0.67–1.01], *p* = 0.060), high BMI (OR 0.74 [95% CI 0.67–0.83], *p* < 0.001), and female gender (OR 14.12 [95% CI 5.91–33.73], *p* < 0.001) increased the probability of low leptin levels (Supplementary Table S4).

## 4. Discussion

**Figure 3:**
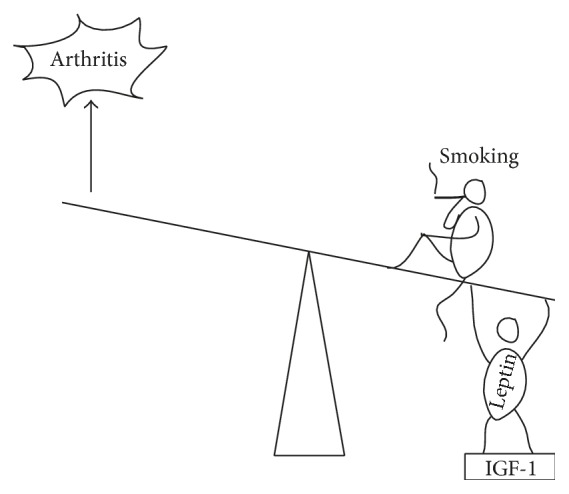
Graphic summary. The combined results obtained on patients with early RA (the Leiden cohort, *n* = 193) and on patients with established and treated RA (the Gothenburg cohort, *n* = 350) indicate that cigarette smoking may be a major cause of acquired IGF1 and leptin deficiency in RA. The multivariate analysis confirmed an association between smoking, IGF1, and leptin, where hypoleptinemia was also shown to be a consequence of higher disease activity. These novel associations are of potential importance for long-term prognosis and in risk assessment for complications and comorbidities in RA.

The results of our study indicate that cigarette smoking may be a major cause of acquired IGF1 and leptin deficiency in RA patients (Figure 3). The multivariate analysis revealed the existing association between these parameters where hypoleptinemia was also shown to be a consequence of higher disease activity. These observations are supported by the reports of an increase in the circulating levels of leptin with a decline of inflammation in RA [34, 35] and with successful antirheumatic treatment. After adjustment to inflammation leptin still showed a negative association with the joint damage [29]. In preclinical setting, both the leptin deficient and leptin receptor deficient mice exhibited a delayed resolution of arthritis [36], and leptin supplementation was reported to ameliorate arthritis [37]. Similar to RA, the importance of leptin has been demonstrated in cardiovascular disease [38–40], malnutrition, and sepsis [41], where low serum levels of leptin are unfavourable for survival. These observations indicate that leptin is not a simple reflection of inflammation being rather engaged in the network of molecular processes in RA pathogenesis.

The physiological role of leptin is currently associated with regulation of the neuroendocrine functions including hypothalamic-growth factor axis and insulin sensitivity with consequences for the energy balance. Stimulation of the growth factor-IGF1 mediated effects makes leptin essential for bone formation acting directly on the bone remodelling cells. In women with hypothalamic amenorrhea, hypoleptinemia is considered the major molecular mechanism of negative bone metabolism in women [42, 43], and leptin supplementation enhanced bone formation [44]. Leptin has been shown to be essential for development of naïve T-cells [45] and immature B-cells supporting intracellular activation of the mechanistic target of rapamycin complex 1 (mTOR) mediated processes. This mechanism explains the controls of leptin over T-cell receptor-dependent functions [46] and the balance between the regulatory T-cells and Th17-cells. Leptin-induced mTOR activation defines a specific molecular and transcriptional signature controlling CD4+ effector T-cell responses [47]. Thus, smoking-induced low levels of IGF1 and leptin may support the aberrant T-cell formation at the preclinical stage of arthritis, in the poor bone remodelling and progressive joint damage in the overt arthritis, and for the early cardiovascular mortality in RA.

There are opposing reports on the local intra-articular effects of leptin and its role in cartilage remodelling. In several experimental studies, leptin is shown to be pr-inflammatory and associated with cartilage damage in antigen-induced arthritis [48–50]. Other studies reported potential protective effect of leptin for the joint cartilage especially in the setting of acute inflammation [37]. Clinical studies followed similar lines; three studies showing high levels of leptin were associated with joint destructive disease [22, 51, 52]. Interestingly, intra-articular levels of leptin were reported to be low compared to serum levels favouring the hypothesis on the cartilage-protective nature of leptin inside the joints. The prospective case-control study showed a negative association between serum leptin and the cartilage changes measured by Larsen score and adjusted for inflammation [29]; a different study reported that high leptin was associated with radiographic progress of joint damage [30]. Three other studies found no association between serum leptin and progress in radiographic changes using longitudinal analysis [26, 27, 53, 54]. The present study suggests a connection between leptin and neuroendocrine processes regulated by IGF-1 in RA since the levels of leptin together with smoking appeared to be the major denominator of IGF1 levels.

The observational design of this study reveals association between leptin and IGF1 without studying their mutual dependence. A bidirectional activation of leptin-IGF1 signalling has been demonstrated experimentally, where leptin competes with IGF1 for binding to IGF1R, and IGF1 in turn activates (phosphorylates) the leptin receptor [24]. In the clinical setting, acquired and congenital hypoleptinemia is followed by a decrease in IGF1 levels and leptin treatment efficiently restores IGF1 levels. The effects of leptin on the levels of IGF1 occur independently of growth hormone, since the increase of IGF1 during growth hormone supplementation was associated with a decrease of leptin [55–57]. The reports above makes leptin a probable mediator of low IGF1 levels in smokers.

Several sources of bias should be considered in this observational study. Stratification of smoking groups is done on the basis of self-reported smoking habits, which may present a source of bias in our study. Accuracy of self-reported smoking varies between studies and is shown to be lower in younger individuals and in socially deprived areas [58]. None of these parameters are applicable in the studied cohorts and enhances confidence in our observations. The use of smokeless tobacco, a common substitute for cigarette smoking in Sweden, is another potential source of bias. Individuals using snuff were consistently excluded from the Gothenburg cohort. The two independent patient cohorts included in the analysis differed with respect to smoking, where the prevalence of former smokers was higher in the Gothenburg cohort. The trichotomization of the whole material permitted the independent analysis of current and former smoking on the low IGF1 levels and showed concordant results. Selection bias may be expected in this study since the Gothenburg cohort has been collected among the methotrexate users where patients with less severe course of RA are underrepresented. However, the cohorts of this study are representative with respect to RF, which permits an assumption of sufficient balance in disease severity. Importantly, the adjustment for RF-positivity did not affect the observed associations between smoking, IGF1 and leptin and makes extrapolation of the results possible on both RF-positive and RF-negative RA populations.

The combination of two independent clinical materials adds certain strength and weakness to the study. On one side, it introduces heterogeneity with respect to disease duration and disease activity, also with respect to experience of different treatment modalities. On the other side, it enhances study power and supports external validity of the obtained results.

## 5. Conclusions

In conclusion, this cross-sectional study identified smoking as an important predictor of low IGF1 and low leptin in RA patients. Importantly, this association existed independently of age, gender, and BMI and may be of importance for the long-term prognosis in RA and development of comorbidities.

## Supplementary Material

Bivariate correlations between BMI and adipokine levels are found in Supplementary Table S1. The complete table of elimination steps from logistic regression models are found in Supplementary Tables S2–S4

## Figures and Tables

**Table 1:** Clinical and demographic characteristics of patients with rheumatoid arthritis in the Gothenburg (Sweden) and Leiden (The Netherlands) study cohorts.

	Gothenburg *n* = 350	Leiden *n* = 193	*p* values
Age, years	57.0 [47.0–63.0]	56.9 [47.4–68.7]	ns^A^
Gender, female/male (%)	256/95 (73%)	129/63 (67%)	ns^B^
BMI, kg/m^2^, *n* = 368	25.7 [22.6–29.0]	25.8 [23.6–27.7]	ns^A^

Disease duration, years	8.0 [3.0–15.0]	0.4 [0.2–0.9]	*p* < 0.001^A^
DAS28, *n* = 229	3.02 [1.90–4.06]	5.17 [4.46–5.91]	*p* < 0.001^A^
VAS-pain, mm, *n* = 373	27.0 [12.0–51.0]	46.0 [28.0–64.3]	*p* < 0.001^A^
RF pos./neg. (%)	228/94 (71%)	115/77 (60%)	*p* = 0.012^B^

Smoking habits, *n* (%)			*p* < 0.001^B^
Smokers	68 (19%)	58 (30%)	
Former smokers	150 (43%)	27 (14%)	
Never smokers	132 (38%)	108 (56%)	

Continuous variables are presented as median [IQR].

BMI: body mass index; DAS: disease activity score; VAS: visual assessment scale; RF: rheumatoid factor

^A^Groups were compared with Mann-Whitney or ^B^Chi-square test.

## References

[1] US Department of Health and Human Services http://www.surgeongeneral.gov/library/reports/50-years-of-progress/full-report.pdf.

[2] Boyer J.-F., Gourraud P.-A., Cantagrel A., Davignon J.-L., Constantin A. (2011). Traditional cardiovascular risk factors in rheumatoid arthritis: a meta-analysis. *Joint Bone Spine*.

[3] Klareskog L., Stolt P., Lundberg K. (2006). A new model for an etiology of rheumatoid arthritis: smoking may trigger HLA-DR (shared epitope)-restricted immune reactions to autoantigens modified by citrullination. *Arthritis & Rheumatism*.

[4] Sugiyama D., Nishimura K., Tamaki K. (2010). Impact of smoking as a risk factor for developing rheumatoid arthritis: a meta-analysis of observational studies. *Annals of the Rheumatic Diseases*.

[5] Di Giuseppe D., Discacciati A., Orsini N., Wolk A. (2014). Cigarette smoking and risk of rheumatoid arthritis: a dose-response meta-analysis. *Arthritis Research & Therapy*.

[6] Sokka T., Hetland M. L., Mäkinen H. (2008). Remission and rheumatoid arthritis: data on patients receiving usual care in twenty-four countries. *Arthritis and Rheumatism*.

[7] Saevarsdottir S., Wedren S., Seddighzadeh M. (2011). Patients with early rheumatoid arthritis who smoke are less likely to respond to treatment with methotrexate and tumor necrosis factor inhibitors: observations from the epidemiological investigation of rheumatoid arthritis and the Swedish Rheumatology Register cohorts. *Arthritis & Rheumatism*.

[8] Hazes J. M. W., Dijkmans B. A. C., Vandenbroucke J. P., De Vries R. R. P., Cats A. (1990). Lifestyle and the risk of rheumatoid arthritis: cigarette smoking and alcohol consumption. *Annals of the Rheumatic Diseases*.

[9] Vesperini V., Lukas C., Fautrel B., Le Loet X., Rincheval N., Combe B. (2013). Association of tobacco exposure and reduction of radiographic progression in early rheumatoid arthritis: results from a French multicenter cohort. *Arthritis Care & Research*.

[10] Finckh A., Dehler S., Costenbader K. H., Gabay C. (2007). Cigarette smoking and radiographic progression in rheumatoid arthritis. *Annals of the Rheumatic Diseases*.

[11] Jiang X., Alfredsson L., Klareskog L., Bengtsson C. (2014). Smokeless tobacco (moist snuff) use and the risk of developing rheumatoid arthritis: results from a case-control study. *Arthritis Care & Research*.

[12] Landin-Wilhelmsen K., Wilhelmsen L., Lappas G. (1994). Serum insulin-like growth factor I in a random population sample of men and women: relation to age, sex, smoking habits, coffee consumption and physical activity, blood pressure and concentrations of plasma lipids, fibrinogen, parathyroid hormone and osteocalcin. *Clinical Endocrinology*.

[13] Faupel-Badger J. M., Berrigan D., Ballard-Barbash R., Potischman N. (2009). Anthropometric correlates of insulin-like growth factor 1 (IGF-1) and IGF binding protein-3 (IGFBP-3) levels by race/ethnicity and gender. *Annals of Epidemiology*.

[14] Puche J. E., Castilla-Cortazar I. (2012). Human conditions of insulin-like growth factor-I (IGF-I) deficiency. *Journal of Translational Medicine*.

[15] Bao Q., Pan J., Qi H. (2014). Aging and age-related diseases—from endocrine therapy to target therapy. *Molecular and Cellular Endocrinology*.

[16] Sheng M. H., Lau K. H., Baylink D. J. (2014). Role of osteocyte-derived insulin-like growth factor i in developmental growth, modeling, remodeling, and regeneration of the bone. *Journal of Bone Metabolism*.

[17] Sandhu M. S., Heald A. H., Gibson J. M., Cruickshank J. K., Dunger D. B., Wareham N. J. (2002). Circulating concentrations of insulin-like growth factor-I and development of glucose intolerance: a prospective observational study. *The Lancet*.

[18] Frystyk J., Ledet T., Møller N., Flyvbjerg A., Ørskov H. (2002). Cardiovascular disease and insulin-like growth factor I. *Circulation*.

[19] Matsumoto T., Tsurumoto T. (2002). Inappropriate serum levels of IGF-I and IGFBP-3 in patients with rheumatoid arthritis. *Rheumatology*.

[20] Bostrom E. A., Svensson M., Andersson S. (2011). Resistin and insulin/insulin-like growth factor signaling in rheumatoid arthritis. *Arthritis & Rheumatism*.

[21] Lemmey A., Maddison P., Breslin A. (2001). Association between insulin-like growth factor status and physical activity levels in rheumatoid arthritis. *The Journal of Rheumatology*.

[22] Toussirot É., Nguyen N. U., Dumoulin G., Aubin F., Cédoz J.-P., Wendling D. (2005). Relationship between growth hormone-IGF-I-IGFBP-3 axis and serum leptin levels with bone mass and body composition in patients with rheumatoid arthritis. *Rheumatology*.

[23] O’Connor J. C., McCusker R. H., Strle K., Johnson R. W., Dantzer R., Kelley K. W. (2008). Regulation of IGF-I function by proinflammatory cytokines: at the interface of immunology and endocrinology. *Cellular Immunology*.

[24] Saxena N. K., Taliaferro-Smith L., Knight B. B. (2008). Bidirectional crosstalk between leptin and insulin-like growth factor-I signaling promotes invasion and migration of breast cancer cells via transactivation of epidermal growth factor receptor. *Cancer Research*.

[25] Jacques C., Holzenberger M., Mladenovic Z. (2012). Proinflammatory actions of visfatin/nicotinamide phosphoribosyltransferase (Nampt) involve regulation of insulin signaling pathway and Nampt enzymatic activity. *The Journal of Biological Chemistry*.

[26] Giles J. T., van der Heijde D. M., Bathon J. M. (2011). Association of circulating adiponectin levels with progression of radiographic joint destruction in rheumatoid arthritis. *Annals of the Rheumatic Diseases*.

[27] Klein-Wieringa I. R., van der Linden M. P. M., Knevel R. (2011). Baseline serum adipokine levels predict radiographic progression in early rheumatoid arthritis. *Arthritis and Rheumatism*.

[28] Klaasen R., Herenius M. M. J., Wijbrandts C. A. (2012). Treatment-specific changes in circulating adipocytokines: a comparison between tumour necrosis factor blockade and glucocorticoid treatment for rheumatoid arthritis. *Annals of the Rheumatic Diseases*.

[29] Rho Y. H., Solus J., Sokka T. (2009). Adipocytokines are associated with radiographic joint damage in rheumatoid arthritis. *Arthritis & Rheumatism*.

[30] Park Y.-J., Cho C.-S., Emery P., Kim W.-U. (2013). LDL cholesterolemia as a novel risk factor for radiographic progression of rheumatoid arthritis: a single-center prospective study. *PLoS ONE*.

[31] Bakker M. F., Cavet G., Jacobs J. W. G. (2012). Performance of a multi-biomarker score measuring rheumatoid arthritis disease activity in the CAMERA tight control study. *Annals of the Rheumatic Diseases*.

[32] Hirata S., Dirven L., Shen Y. (2013). A multi-biomarker score measures rheumatoid arthritis disease activity in the best study. *Rheumatology*.

[33] Yusuf E., Ioan-Facsinay A., Bijsterbosch J. (2011). Association between leptin, adiponectin and resistin and long-term progression of hand osteoarthritis. *Annals of the Rheumatic Diseases*.

[34] Popa C., Netea M. G., Radstake T. R. D. S., Van Riel P. L., Barrera P., Van Der Meer J. W. M. (2005). Markers of inflammation are negatively correlated with serum leptin in rheumatoid arthritis. *Annals of the Rheumatic Diseases*.

[35] Chen C.-Y., Tsai C.-Y., Lee P.-C., Lee S.-D. (2013). Long-term etanercept therapy favors weight gain and ameliorates cachexia in rheumatoid arthritis patients: roles of gut hormones and leptin. *Current Pharmaceutical Design*.

[36] Bernotiene E., Palmer G., Talabot-Ayer D., Szalay-Quinodoz I., Aubert M. L., Gabay C. (2004). Delayed resolution of acute inflammation during zymosan-induced arthritis in leptin-deficient mice. *Arthritis Research & Therapy*.

[37] Hultgren O. H., Tarkowski A. (2001). Leptin in septic arthritis: decreased levels during infection and amelioration of disease activity upon its adminstration. *Arthritis Research*.

[38] Unger R. H. (2005). Hyperleptinemia: protecting the heart from lipid overload. *Hypertension*.

[39] Smith C. C. T., Mocanu M. M., Davidson S. M., Wynne A. M., Simpkin J. C., Yellon D. M. (2006). Leptin, the obesity-associated hormone, exhibits direct cardioprotective effects. *British Journal of Pharmacology*.

[40] Morita Y., Maeda K., Kondo T. (2013). Impact of adiponectin and leptin on long-term adverse events in Japanese patients with acute myocardial infarction. Results from the Nagoya Acute Myocardial Infarction Study (NAMIS). *Circulation Journal*.

[41] Bracho-Riquelme R. L., Loera-Castañeda V., Torres-Valenzuela A., Loera-Castañeda G. A., Sánchez-Ramírez J. P. (2011). Leptin and leptin receptor polymorphisms are associated with poor outcome (death) in patients with non-appendicular secondary peritonitis. *Critical Care*.

[42] Welt C. K., Chan J. L., Bullen J. (2004). Recombinant human leptin in women with hypothalamic amenorrhea. *The New England Journal of Medicine*.

[43] Chou S. H., Chamberland J. P., Liu X. (2011). Leptin is an effective treatment for hypothalamic amenorrhea. *Proceedings of the National Academy of Sciences of the United States of America*.

[44] Bartell S. M., Rayalam S., Ambati S. (2011). Central (ICV) leptin injection increases bone formation, bone mineral density, muscle mass, serum IGF-1, and the expression of osteogenic genes in leptin-deficient ob/ob mice. *Journal of Bone and Mineral Research*.

[45] Lord G. M., Matarese G., Howard J. K., Baker R. J., Bloom S. R., Lechler R. I. (1998). Leptin modulates the T-cell immune response and reverses starvation- induced immunosuppression. *Nature*.

[46] Farooqi I. S., Matarese G., Lord G. M. (2002). Beneficial effects of leptin on obesity, T cell hyporesponsiveness, and neuroendocrine/metabolic dysfunction of human congenital leptin deficiency. *The Journal of Clinical Investigation*.

[47] Procaccini C., De Rosa V., Galgani M. (2012). Leptin-induced mTOR activation defines a specific molecular and transcriptional signature controlling CD4^+^ effector T cell responses. *The Journal of Immunology*.

[48] Busso N., So A., Chobaz-Péclat V. (2002). Leptin signaling deficiency impairs humoral and cellular immune responses and attenuates experimental arthritis. *Journal of Immunology*.

[49] Otvos L., Shao W.-H., Vanniasinghe A. S. (2011). Toward understanding the role of leptin and leptin receptor antagonism in preclinical models of rheumatoid arthritis. *Peptides*.

[50] Deng J., Liu Y., Yang M. (2012). Leptin exacerbates collagen-induced arthritis via enhancement of Th17 cell response. *Arthritis & Rheumatism*.

[51] Bokarewa M., Bokarew D., Hultgren O., Tarkowski A. (2003). Leptin consumption in the inflamed joints of patients with rheumatoid arthritis. *Annals of the Rheumatic Diseases*.

[52] Olama S. M., Senna M. K., Elarman M. (2012). Synovial/serum leptin ratio in rheumatoid arthritis: the association with activity and erosion. *Rheumatology International*.

[53] Giles J. T., Allison M., Bingham C. O., Scott W. M., Bathon J. M. (2009). Adiponectin is a mediator of the inverse association of adiposity with radiographic damage in rheumatoid arthritis. *Arthritis Care and Research*.

[54] Meyer M., Sellam J., Fellahi S. (2013). Serum level of adiponectin is a surrogate independent biomarker of radiographic disease progression in early rheumatoid arthritis: results from the ESPOIR cohort. *Arthritis Research & Therapy*.

[55] Tillmann V., Patel L., Gill M. S. (2000). Monitoring serum insulin-like growth factor-I (IGF-I), IGF binding protein-3 (IGFBP-3), IGF-I/IGFBP-3 molar ratio and leptin during growth hormone treatment for disordered growth. *Clinical Endocrinology*.

[56] Ciresi A., Amato M. C., Criscimanna A. (2007). Metabolic parameters and adipokine profile during GH replacement therapy in children with GH deficiency. *European Journal of Endocrinology/European Federation of Endocrine Societies*.

[57] Su P.-H., Chen J.-Y., Yu J.-S., Chen S.-J., Yang S.-F. (2011). Leptin expression and leptin receptor gene polymorphisms in growth hormone deficiency patients. *Human Genetics*.

[58] Connor Gorber S., Schofield-Hurwitz S., Hardt J., Levasseur G., Tremblay M. (2009). The accuracy of self-reported smoking: a systematic review of the relationship between self-reported and cotinine-assessed smoking status. *Nicotine & Tobacco Research*.

