# Supplementary material for: Smoking Functions as a Negative Regulator of IGF1 and Impairs Adipokine Network in Patients with Rheumatoid Arthritis
Source: Mediators Inflamm. 2016 Mar 3;2016:3082820. doi: 10.1155/2016/3082820 (PMC4794568; doi:10.1155/2016/3082820)
Supplement: Supplementary file 1 — Bivariate correlations between BMI and adipokine levels are found in Supplementary Table S1. The complete table of elimination steps from logistic regression models are found in Supplementary Tables S2–S4 [file 3082820.f1.docx]

**Supplementary table S1**

Correlations of serum levels of IGF1 and adipokines to BMI

|  | Spearman correlations to BMI, kg/m^2^ |
| --- | --- |
| IGF1, ng/ml | Rho=-0.053, ns |
| Leptin, ng/ml | Rho=0.467, p<0.0001 |
| Adiponectin; µg/ml | Rho=-0.218, p<0.0001 |
| Resistin, ng/ml | Rho=0.023, ns |
| Visfatin, ng/ml | Rho=0.107, p=0.042 |

**Supplementary Table S2**

**Binary logistic regression A. Dependent variable: IGF1 low – adipokine levels**

N=515

Smoking (1) = present smoking, Smoking (2) = normer smoking compared to never smoking

Gender (1) = female compared to male

B S.E. Wald df Sig. Exp(B) 95% C.I.for EXP(B)

Lower Upper

Step 1a Gender(1) .314 .251 1.572 1 .210 1.370 .838 2.239

Age .006 .008 .480 1 .489 1.006 .990 1.021

tertVISF -.209 .129 2.643 1 .104 .811 .630 1.044

tertAdipo .178 .133 1.787 1 .181 1.195 .920 1.552

trtLEPT -.253 .137 3.422 1 .064 .776 .594 1.015

tertRES .171 .129 1.762 1 .184 1.186 .922 1.527

smoking 9.527 2 .009

smoking(1) -.595 .240 6.123 1 .013 .552 .344 .884

smoking(2) .151 .258 .344 1 .558 1.163 .702 1.927

Constant -.934 .653 2.049 1 .152 .393

Step 2a Gender(1) .331 .249 1.756 1 .185 1.392 .854 2.269

tertVISF -.196 .127 2.372 1 .124 .822 .641 1.055

tertAdipo .193 .132 2.156 1 .142 1.213 .937 1.570

trtLEPT -.248 .137 3.303 1 .069 .780 .597 1.020

tertRES .168 .129 1.708 1 .191 1.183 .919 1.522

smoking 10.103 2 .006

smoking(1) -.619 .238 6.769 1 .009 .538 .338 .858

smoking(2) .136 .257 .282 1 .595 1.146 .693 1.895

Constant -.682 .540 1.594 1 .207 .505

Step 3a Gender(1) .336 .249 1.816 1 .178 1.399 .859 2.279

tertVISF -.153 .123 1.552 1 .213 .858 .675 1.092

tertAdipo .187 .131 2.028 1 .154 1.205 .932 1.558

trtLEPT -.241 .136 3.118 1 .077 .786 .602 1.027

smoking 10.299 2 .006

smoking(1) -.612 .237 6.642 1 .010 .542 .340 .864

smoking(2) .160 .255 .390 1 .532 1.173 .711 1.935

Constant -.441 .508 .756 1 .385 .643

Step 4a Gender(1) .302 .247 1.493 1 .222 1.353 .833 2.197

tertAdipo .178 .130 1.865 1 .172 1.195 .925 1.543

trtLEPT -.251 .136 3.412 1 .065 .778 .596 1.015

smoking 10.142 2 .006

smoking(1) -.613 .237 6.690 1 .010 .542 .340 .862

smoking(2) .145 .255 .325 1 .569 1.156 .702 1.905

Constant -.692 .467 2.193 1 .139 .501

Step 5a tertAdipo .127 .123 1.069 1 .301 1.136 .892 1.446

trtLEPT -.318 .124 6.546 1 .011 .727 .570 .928

smoking 11.265 2 .004

smoking(1) -.637 .236 7.306 1 .007 .529 .333 .839

smoking(2) .157 .254 .383 1 .536 1.170 .711 1.925

Constant -.358 .378 .897 1 .344 .699

Step 6a trtLEPT -.321 .124 6.687 1 .010 .725 .569 .925

smoking 10.563 2 .005

smoking(1) -.617 .235 6.910 1 .009 .540 .341 .855

smoking(2) .138 .253 .298 1 .585 1.148 .699 1.885

Constant -.100 .283 .126 1 .723 .904

a Variable(s) entered on step 1: Gender, Age, tertVISF, tertAdipo, trtLEPT, tertRES, smoking.

**Supplementary table S3**

**Binary logistic regression B. Dependent variable: IGF1 low – clinical variables**

n=246

Smoking (1) = present smoking, Smoking (2) = normer smoking compared to never smoking

Gender (1) = female compared to male

B S.E. Wald df Sig. Exp(B) 95% C.I.for EXP(B)

Lower Upper

Step 1a Gender(1) .359 .378 .901 1 .342 1.432 .682 3.007

Age .000 .016 .000 1 .993 1.000 .970 1.031

smoking 11.603 2 .003

smoking(1) -.832 .406 4.212 1 .040 .435 .196 .963

smoking(2) .726 .395 3.375 1 .066 2.067 .953 4.485

DD_yr .023 .018 1.615 1 .204 1.023 .987 1.061

RF_seropos(1) .269 .401 .449 1 .503 1.309 .596 2.874

DAS28 -.103 .107 .928 1 .335 .902 .732 1.112

VASpain -.011 .007 2.273 1 .132 .989 .975 1.003

BMI .008 .035 .057 1 .811 1.008 .942 1.080

Constant -.974 1.175 .687 1 .407 .378

Step 2a Gender(1) .359 .375 .915 1 .339 1.432 .686 2.987

smoking 11.768 2 .003

smoking(1) -.832 .398 4.359 1 .037 .435 .199 .950

smoking(2) .726 .395 3.387 1 .066 2.067 .954 4.480

DD_yr .023 .018 1.676 1 .196 1.023 .988 1.060

RF_seropos(1) .269 .400 .452 1 .501 1.309 .597 2.868

DAS28 -.103 .106 .949 1 .330 .902 .733 1.110

VASpain -.011 .007 2.278 1 .131 .989 .975 1.003

BMI .008 .034 .059 1 .809 1.008 .943 1.078

Constant -.979 .987 .984 1 .321 .376

Step 3a Gender(1) .368 .373 .971 1 .324 1.445 .695 3.002

smoking 11.726 2 .003

smoking(1) -.836 .398 4.411 1 .036 .433 .199 .946

smoking(2) .712 .390 3.331 1 .068 2.039 .949 4.380

DD_yr .023 .018 1.680 1 .195 1.023 .988 1.060

RF_seropos(1) .277 .399 .479 1 .489 1.319 .603 2.885

DAS28 -.102 .106 .932 1 .334 .903 .734 1.111

VASpain -.011 .007 2.228 1 .136 .989 .975 1.003

Constant -.773 .499 2.396 1 .122 .462

Step 4a Gender(1) .342 .371 .850 1 .357 1.408 .680 2.916

smoking 12.020 2 .002

smoking(1) -.843 .398 4.491 1 .034 .430 .197 .939

smoking(2) .724 .389 3.457 1 .063 2.062 .962 4.424

DD_yr .021 .017 1.388 1 .239 1.021 .986 1.056

DAS28 -.100 .106 .901 1 .342 .905 .735 1.113

VASpain -.011 .007 2.532 1 .112 .989 .975 1.003

Constant -.671 .476 1.994 1 .158 .511

Step 5a smoking 12.646 2 .002

smoking(1) -.870 .396 4.824 1 .028 .419 .193 .911

smoking(2) .728 .388 3.517 1 .061 2.072 .968 4.435

DD_yr .020 .017 1.382 1 .240 1.021 .986 1.056

DAS28 -.100 .105 .908 1 .341 .904 .736 1.112

VASpain -.011 .007 2.324 1 .127 .989 .976 1.003

Constant -.604 .468 1.665 1 .197 .547

Step 6a smoking 12.516 2 .002

smoking(1) -.879 .396 4.940 1 .026 .415 .191 .901

smoking(2) .702 .386 3.304 1 .069 2.018 .947 4.302

DD_yr .023 .017 1.796 1 .180 1.023 .990 1.058

VASpain -.013 .007 3.514 1 .061 .987 .974 1.001

Constant -.880 .370 5.659 1 .017 .415

Step 7a smoking 12.519 2 .002

smoking(1) -.944 .392 5.799 1 .016 .389 .180 .839

smoking(2) .626 .380 2.714 1 .099 1.869 .888 3.935

VASpain -.014 .007 4.072 1 .044 .986 .973 1.000

Constant -.596 .300 3.945 1 .047 .551

a Variable(s) entered on step 1: Gender, Age, smoking, DD_yr, RF_seropos, DAS28, VASpain, BMI.

**Supplementary Table S4**

**Binary logistic regression C. Dependent variable: Leptin low – clinical variables**

n=246

Smoking (1) = present smoking, Smoking (2) = normer smoking compared to never smoking

Gender (1) = female compared to male

B S.E. Wald df Sig. Exp(B) 95% C.I.for EXP(B)

Lower Upper

Step 1a Gender(1) 2.682 .456 34.551 1 .000 14.616 5.976 35.745

Age .009 .015 .322 1 .571 1.009 .979 1.039

smoking .067 2 .967

smoking(1) .049 .396 .015 1 .902 1.050 .483 2.284

smoking(2) -.075 .465 .026 1 .873 .928 .373 2.310

DD_yr -.007 .020 .136 1 .712 .993 .955 1.032

RF_seropos(1) .002 .453 .000 1 .997 1.002 .412 2.435

DAS28 -.165 .115 2.061 1 .151 .848 .677 1.062

VASpain -.009 .008 1.334 1 .248 .991 .976 1.006

BMI -.303 .057 28.267 1 .000 .739 .661 .826

Constant 6.539 1.515 18.626 1 .000 691.252

Step 2a Gender(1) 2.682 .454 34.919 1 .000 14.613 6.004 35.568

Age .009 .015 .322 1 .571 1.009 .979 1.039

smoking .068 2 .967

smoking(1) .049 .395 .015 1 .902 1.050 .484 2.277

smoking(2) -.074 .463 .026 1 .872 .928 .374 2.302

DD_yr -.007 .020 .141 1 .707 .993 .955 1.031

DAS28 -.165 .115 2.061 1 .151 .848 .677 1.062

VASpain -.009 .008 1.356 1 .244 .991 .976 1.006

BMI -.303 .057 28.573 1 .000 .739 .661 .825

Constant 6.539 1.515 18.635 1 .000 691.326

Step 3a Gender(1) 2.676 .454 34.794 1 .000 14.525 5.970 35.340

Age .008 .015 .295 1 .587 1.008 .979 1.038

DD_yr -.007 .019 .146 1 .703 .993 .956 1.031

DAS28 -.166 .114 2.109 1 .146 .847 .678 1.060

VASpain -.009 .008 1.419 1 .234 .991 .976 1.006

BMI -.303 .056 28.834 1 .000 .739 .662 .825

Constant 6.571 1.436 20.929 1 .000 714.200

Step 4a Gender(1) 2.676 .453 34.853 1 .000 14.523 5.974 35.306

Age .007 .014 .222 1 .637 1.007 .979 1.036

DAS28 -.159 .113 1.993 1 .158 .853 .684 1.064

VASpain -.009 .008 1.345 1 .246 .991 .976 1.006

BMI -.302 .056 28.823 1 .000 .740 .663 .826

Constant 6.516 1.428 20.821 1 .000 675.796

Step 5a Gender(1) 2.704 .450 36.113 1 .000 14.938 6.184 36.082

DAS28 -.153 .112 1.873 1 .171 .858 .689 1.068

VASpain -.009 .008 1.506 1 .220 .991 .976 1.006

BMI -.296 .055 29.241 1 .000 .744 .668 .828

Constant 6.733 1.356 24.666 1 .000 839.282

Step 6a Gender(1) 2.648 .444 35.543 1 .000 14.123 5.914 33.727

DAS28 -.198 .105 3.549 1 .060 .821 .668 1.008

BMI -.297 .055 29.503 1 .000 .743 .667 .827

Constant 6.606 1.348 24.020 1 .000 739.750

a Variable(s) entered on step 1: Gender, Age, smoking, DD_yr, RF_seropos, DAS28, VASpain, BMI.
